# Resistant and Resilient mutations in protection against familial Alzheimer’s disease: learning from nature

**DOI:** 10.1186/s13024-023-00626-3

**Published:** 2023-06-01

**Authors:** Diego Sepulveda-Falla

**Affiliations:** Molecular Neuropathology of Alzheimer’s Disease, Institute of Neuropathology, University Medical Center Hamburg-Eppendorf, Hamburg, Germany

Alzheimer’s disease (AD) is the most common cause of dementia and a multifactorial disorder affecting around 50 million people worldwide. It is characterized by progressive cognitive impairment eventually leading to death. Postmortem findings of AD pathological hallmarks remain as the main criteria for a definitive diagnosis, despite considerable advances in diagnostic biomarkers. The identification of causative and risk genes, together with the study of their possible pathophysiological effects, have provided the best clues for understanding the disease. Amyloid Precursor Protein (*APP*), Presenilin-1 (*PSEN1*), and Presenilin-2 (*PSEN2*) mutations have been identified as causative, while Apolipoprotein E (*APOE*) haplotype 4 (*APOE4*) increases the risk of developing AD 3- to fourfold [1]. Conversely, heterozygous *APOE* haplotype 2 (*APOE2*) carriers’ lifetime risk of developing AD is decreased by half. Rarer *APOE* variants such as *APOE3* V236E (Jacksonville) and *APOE4* R251G have shown evidence of decreased risk for AD. *APOE3*-Jacksonville reduces ApoE self-aggregation, favoring lipid association and reducing amyloid load and toxicity [2]. A homozygous carrier for the *APOE3 R136S* mutation (*APOE3* Christchurch, *APOEch*) has shown to be protected against AD for three decades despite carrying *PSEN1 E280A* mutation [3]. Finally, *APP* protective mutation (A673T) improved cognitive performance, and decreased Aβ peptide pathology [4].

Natural protection to AD is currently defined by two paths, “Resistance” and “Resilience”. “Resistance” refers to cases when the subject shows less than expected cognitive impairment, and less than expected amyloid beta peptide (Aβ) or hyperphosphorylated Tau protein (pTau) pathologies, despite a probable AD diagnosis. In AD Resistance, the mechanisms of protection interfere successfully with the pathological chain of events that leads to deposition of these proteins, resulting in preserved cognition. “Resilience”, refers to the cases where the full spectra of pathological events typically associated with full-fledged AD dementia occurs, including severe deposition of Aβ and pTau, but cognitive performance is preserved—the brain withstands the pathology and remains functional. Both forms of protection have been identified in sporadic AD patients [5]. However, Resistance could be a regular progressing AD interrupted by death. Or Resilience could be a slower progressing case, also interrupted. AD protection researchers have attempted to control for these possibilities in their studies, with variable success [6].

The *PSEN1 E280A* Colombian kindred is the largest known familial AD (FAD) cohort. It comprises around 6000 individuals, including 1200 living carriers. Several generations of carriers have been studied longitudinally for more than 30 years [7]. The size of the cohort has allowed several crucial discoveries in the clinical progression, biomarker behavior and pathophysiology of FAD [8]. This cohort shows a founder effect, with family members sharing a similar diet and other environmental factors, making it the most uniform human model possible for the study of AD. However, they show high variability of clinical profiles underlying the amnesic dementia phenotype, including seizures, language impairment, headache, and motor symptoms [7].

Dementia age of onset is an established parameter to measure disease severity in FAD. It has been attempted to predict it in FAD patients, or to characterize it as a feature associated with mutation localization in the sequence of the *PSEN1* gene [9]. Such type of studies, however, have been limited by the low number of cases for each of the mutations or families involved. The large *PSEN1 E280A* cohort shows a median age of MCI onset of 44 years old, and dementia onset of 49 years old. However, the clearest indicator for disease heterogeneity in FAD, as an effect of individual modifying factors, is that age of dementia onset ranges from 37 to 77 years old among *PSEN1 E280A* carriers (Fig. 1A) [8]. It is also the first indicator of possible protective mechanisms against FAD in this population, recently associated with less severe pTau pathology and delayed disease onset [8, 10].

**Fig. 1:**
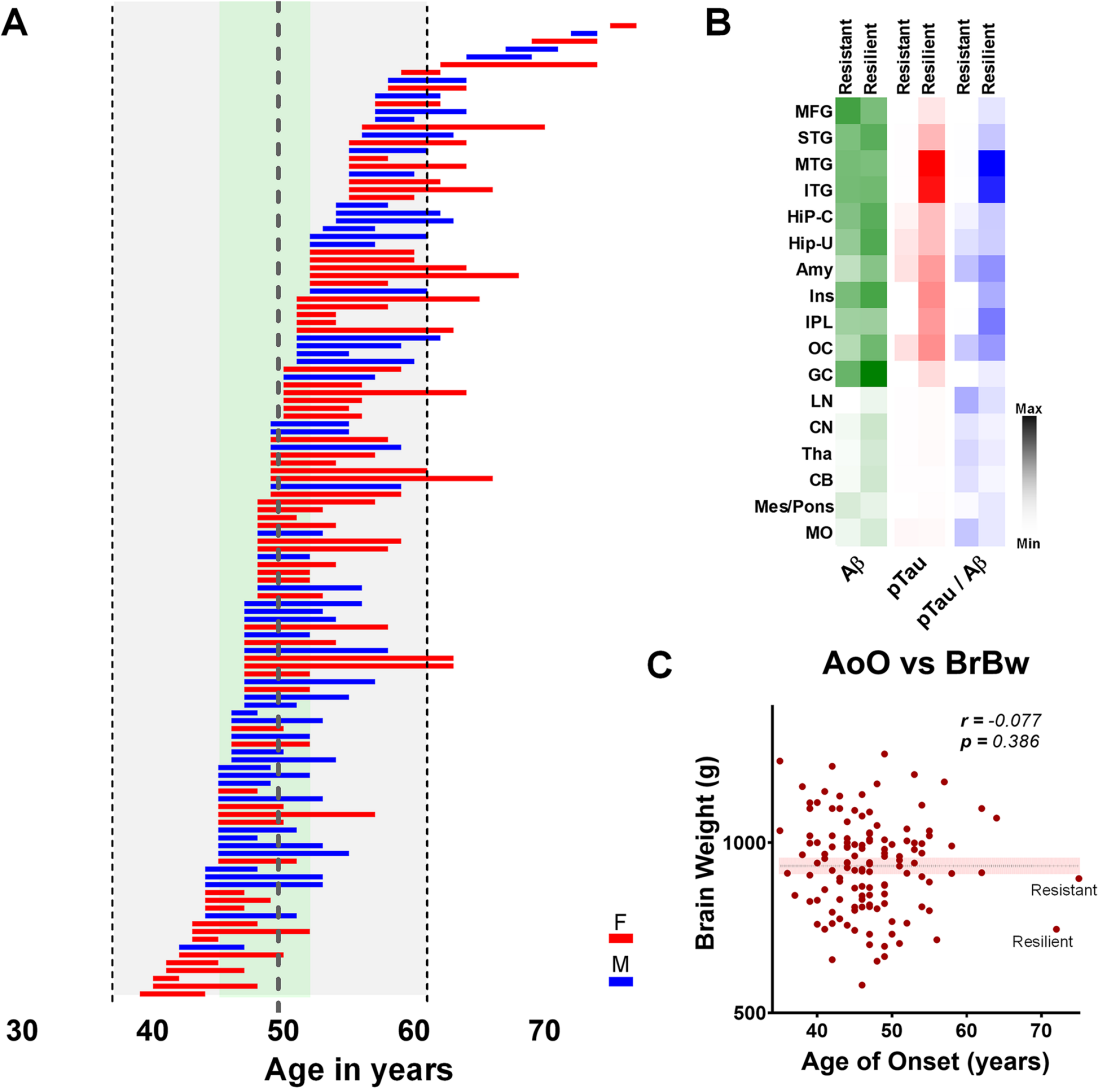
**A** Bar graph depicting age of onset and disease duration of 125 *PSEN1 E280A* cases (females = red, males = blue). The thick dotted line represents average age of onset (49.64 years old), thinner dotted lines represent -2 and + 2 standard deviations (± 12.04 years) from it, and the area between age of onset quartiles 25% and 75% is shaded in pale green. **B** heatmaps depicting normalized values taken from [10] and [11], for signal intensity from Aβ (green), pTau (red) and the ratio of pTau signal over Aβ signal (blue). “Resistant” refers to the *PSEN1 E280A APOEch* carrier, and “Resilience” to the RELN-COLBOS one. Studied areas were: middle frontal gyrus (MFG), superior temporal gyrus (STG), middle temporal gyrus (MTG), inferior temporal gyrus (ITG), hippocampus – CA (Hip-C), hippocampus—uncus (Hip-U), amygdala (Amy), insula (Ins), inferior parietal lobe (IPL), occipital cortex (OC), gyrus cinguli (GC), lenticular nucleus (LN), caudate nucleus (CN), thalamus (Tha), cerebellum (CB), mesencephalon / Pons (Mes/pons), medulla oblongata (MO). **C** scatterplot of brain weight (BrBw) at time of death according to age of onset of dementia (AoO) in 125 *PSEN1 E280A* cases. There is no correlation between these two variables. Note the wide distribution of BrBw, and the difference between BrBw in both protected cases

Previously, a *PSEN1 E280A* carrier also carrying two copies for the APOEch was reported [3]. This patient presented MCI onset at 72 years of age, dementia onset at 75 years of age, and died two years later. Tau Positron Emission Tomography (PET) showed less burden in temporal and parietal cortices compared to other *PSEN1 E280A* carriers. Postmortem analysis showed extended Aβ pathology in all cortices and some subcortical structures. Meanwhile, the frontal cortex was mostly spared for pTau pathology, with other cortical structures showing higher pTau pathology, and strong pathology in amygdala and occipital cortex [10] (Fig. 1B). As a *PSEN1 E280A* carrier, the age of dementia onset was expected to take place at least 25 years earlier and shown more extended generalized cortical pTau pathology. This case is an ideal example for AD Resistance.

More recently, another strongly protected case was identified among *PSEN1 E280A* carriers, carrying also a heterozygous mutation H3447R in the Reelin gene (*RELN*), denominated *RELN-COLBOS* [11]. Dementia onset was at 72 years of age with a disease duration of two years. Tau PET imaging showed limited pTau pathology in the entorhinal cortex (EC). Postmortem analysis showed that cortical structures were more affected by pTau pathology in the *RELN-COLBOS* case compared with the *APOEch* case (Fig. 1B). In fact, it showed severe brain atrophy (Fig. 1C), together with severe Aβ and pTau pathologies. Notably, the EC in the *RELN-COLBOS* case showed less Aβ and pTau pathology and higher neuronal density in EC supra and infragranular layers, compared to any other AD case evaluated in that study, including the *APOEch* carrier. It has been suggested that Reelin positive cells in neuronal layer II of the EC play a crucial role in the early stages of AD. These neurons show high energy demand, making them more vulnerable to known AD-related events, such as mitochondrial dysfunction [12]. Any possible mechanisms of protection in the *RELN-COLBOS* case did not modify pathology presentation, nor general neurodegeneration. However, it did preserve neuronal pathways necessary for maintaining cognitive performance far beyond expectations for a patient belonging to this cohort. Thus, due to the pathological evidence presented in this case, this is a better example of AD Resilience.

Cohort size and disease heterogeneity in the *PSEN1 E280A* allowed for two outstanding examples of protection against AD dementia. Both mutated proteins, ApoE and Reelin, share receptors (ApoE Receptor 2 and Very Low-density Lipoprotein Receptor) and molecular pathways, and they possibly share mechanistic effects such as tau phosphorylation modulation via GSK3β [13]. However, ApoE is by far a more widely expressed molecule that might explain resistance to AD pathology, driven by global high *APOE* expression in astrocytes and microglia [10]. On the other hand, Reelin expresses mainly in very specific brain regions and cell populations, leading to a localized protective effect and favoring the survival of key neuronal pathways that result in delayed onset of dementia, regardless AD pathology severity, suggesting a Resilient phenotype.

Why have these mutations not been previously identified as protective for AD? Would the effect be the same in sporadic AD cases? Given the multifactorial nature of AD, several possible protective mechanisms are bound to occur. Prospectively, new protected cases will be identified in the *PSEN1 E280A* cohort, whether carrying the same mutated genes or others. For successful identification also in sporadic AD, several conditions need to be satisfied: a. the putative strength of the protection should overcome other risk and causative factors, b. other possible neurodegenerative comorbidities can also be neutralized by the same protective mechanism, c. both protection and disease onset should occur at an age in which they can be still clinically identified. Regarding therapeutic developments, some aspects should be taken in consideration. *APOEch* heterozygous AD cases, sporadic or familial, do not show the same degree of protection than the *APOEch PSEN1 E280A* case [14], hinting towards a dose dependent effect that might be needed to be kept for a successful therapeutic effect. More importantly, the effects of all mutations present in these two unique cases are acting from birth, therefore it is possible that the high degree of protection is achieved by a cumulative effect throughout life.

The *RELN-COLBOS* Resilient case points towards a more intriguing possibility in AD. By being cognitively unimpaired for several decades while having severe AD pathology, it suggests that the direct cause of AD dementia is not necessarily the pathological events that lead to its visible hallmarks. More likely, there are key neuronal populations that are essential for the maintenance of cognitive function, including memory, which might be vulnerable to the early toxic environment of what will become visible AD pathology. Some findings support this notion, for instance the identification of RORB positive neurons vulnerable to pTau pathology [15]. Thus, perhaps AD pathological cascades have parallel effects, one global and eventually visible as AD pathology, and one local on specific vulnerable neurons linked to cognition. The *RELN-COLBOS PSEN1 E280A* case suggests that protecting key neuronal populations might suffice as a therapeutic strategy against AD, provided that it could be administered before these neuronal populations are affected. The unequivocal identification of these neurons might be the next lesson that nature is presenting us for untangling the AD problem.

## Data Availability

Not applicable.

## Abbreviations

AD: Alzheimer’s Disease
Aβ: Amyloid beta peptide
APOE: Apolipoprotein E gene
ApoE: Apolipoprotein E
APOEch: APOE3 Christchurch
APP: Amyloid Precursor Protein
EC: Entorhinal Cortex
MCI: Mild cognitive impairment
PET: Positron Emission Tomography
PSEN1: Presenilin-1
pTau: Hyperphosphorylated Tau protein
RELN: Reelin gene
RELN-COLBOS: Reelin H3447R mutation
